# Genetic insights into PHARC syndrome: identification of a novel frameshift mutation in *ABHD12*

**DOI:** 10.1186/s12920-023-01682-w

**Published:** 2023-10-06

**Authors:** Ahmad Daneshi, Masoud Garshasbi, Mohammad Farhadi, Khalil Ghasemi Falavarjani, Mohammad Vafaee-Shahi, Navid Almadani, MohammadSina Zabihi, Mohammad Amin Ghalavand, Masoumeh Falah

**Affiliations:** 1 ENT and Head and Neck Research Center and Department, The Five Senses Health Institute, School of Medicine, Hazrat Rasoul Akram Hospital, Iran University of Medical Sciences, Tehran, Iran; 2 Department of Medical Genetics, Faculty of Medical Sciences, Tarbiat Modares University, Tehran, Iran; 3 Eye Research Centre, Five Senses Health Institute, School of Medicine, Hazrat Rasoul Akram Hospital, Iran University of Medical Sciences, Tehran, Iran; 4 Stem Cell and Regenerative Medicine Research Center, School of Medicine, Iran University of Medical Sciences, Tehran, Iran; 5 Pediatric Growth and Development Research Center, Institute of Endocrinology and metabolism, School of Medicine, Iran University of Medical Sciences, Tehran, Iran; 6 Department of Genetics, Reproductive Biomedicine Research Center, Royan Institute for Reproductive Biomedicine, ACECR, Tehran, Iran

**Keywords:** Hearing loss, Polyneuropathy, Retinitis pigmentosa, Ataxia, Cataract, PHARC, Neurodegenerative, *ABHD12*, Whole-exome sequencing, Endocannabinoid

## Abstract

**Background:**

Mutations in *ABHD12* (OMIM: 613,599) are associated with polyneuropathy, hearing loss, ataxia, retinitis pigmentosa, and cataract (PHARC) syndrome (OMIM: 612674), which is a rare autosomal recessive neurodegenerative disease. PHARC syndrome is easily misdiagnosed as other neurologic disorders, such as retinitis pigmentosa, Charcot-Marie-Tooth disease, and Refsum disease, due to phenotype variability and slow progression. This paper presents a novel mutation in *ABHD12* in two affected siblings with PHARC syndrome phenotypes. In addition, we summarize genotype-phenotype information of the previously reported patients with *ABHD12* mutation.

**Methods:**

Following a thorough medical evaluation, whole-exome sequencing was done on the proband to look for potential genetic causes. This was followed by confirmation of identified variant in the proband and segregation analysis in the family by Sanger sequencing. The variants were interpreted based on the American College of Medical Genetics and Genomics (ACMG) guidelines.

**Results:**

A novel pathogenic homozygous frameshift variant, NM_001042472.3:c.601dup, p.(Val201GlyfsTer4), was identified in exon 6 of *ABHD12* (ACMG criteria: PVS1 and PM2, PM1, PM4, PP3, and PP4). Through Sanger sequencing, we showed that this variant is co-segregated with the disease in the family. Further medical evaluations confirmed the compatibility of the patients’ phenotype with PHARC syndrome.

**Conclusions:**

Our findings expand the spectrum of mutations in the *ABHD12* and emphasize the significance of multidisciplinary diagnostic collaboration among clinicians and geneticists to solve the differential diagnosis of related disorders. Moreover, a summary based on mutations found so far in the *ABHD12* gene did not suggest a clear genotype-phenotype correlation for PHARC syndrome.

## Introduction

Concurrent impairments of the essential senses of hearing and vision greatly influence affected individuals’ quality of life and often result in morbidity and mortality [1–4]. This accompaniment accounts for nearly 0.015% of the general population, with patients under 18 years of age making up 5.7% of this group [5]. Among diverse etiological reasons for these impairments, heritable factors are estimated to be responsible for 27% of cases [5]. Usher syndrome has the highest frequency among such impairments [5, 6]; other syndromes include PHARC (polyneuropathy, hearing loss, ataxia, retinitis pigmentosa, and cataract) syndrome (OMIM: 612674), Heimler syndrome 1 (OMIM: 234580), Alstrom syndrome (OMIM: 203800), Bardet-Biedel syndrome (OMIM: 209900), and Cone-rod dystrophy and hearing loss 1 (OMIM: 617236).

PHARC syndrome is an autosomal recessive neurodegenerative disease influencing the peripheral and central nervous systems. Its name is taken from its significant features, including polyneuropathy, hearing loss, ataxia, retinitis pigmentosa (RP), and cataract [7], although not all of these features necessarily manifest at the initial presentation [7, 8]. Some patients show only some of these symptoms for years so affected individuals are usually misdiagnosed with other neurodegenerative diseases like Usher syndrome, RP, Refsum, Charcot-Marie-Tooth, and mitochondrial diseases [8, 9]. Genetic testing can lead to a definitive diagnosis by differentiating between these similar syndromes.

Loss of function mutations in the *ABDH12* gene (OMIM: 613599) cause PHARC syndrome. This gene contains 13 coding exons on chromosome 20 and translates to an α/βhydrolase domain-containing 12 (ABHD12) protein. The ABHD12 protein is a kind of enzyme that participates in lipid metabolism by catalyzing 2-arachidonoyl glycerol (2-AG) [7]. 2-AG, as the main endocannabinoid lipid transmitter, acts in neuroinflammation and synaptic plasticity. The endocannabinoid system participates in different biological processes, for instance, neurotransmission, inflammation, mood, appetite, pain appreciation, and addiction behavior [10]. ABHD12 is expressed in different mouse tissues, but the highest expression has been observed in microglia and macrophages, especially in the brain [7]. Since a single functional copy of *ABHD12* makes sufficient enzyme activity therefore, heterozygous carriers do not present any clinical features [11, 12].

The current challenge of diagnosing PHARC syndrome makes it essential to investigate its clinical and genetic features. Increasing data in these areas can expand the current knowledge about its onset, the existence of genotype-phenotype correlations, and the natural history of PHARC syndrome; it may also help introduce new potential treatment strategies.

This report presents the clinical manifestation of two affected individuals from a consanguineous Iranian family with mild sensory symptoms, progressive hearing impairment, cataract, and RP. Whole-exome sequencing (WES), followed by segregation analysis, confirmed a novel biallelic mutation in *ABHD12*. We also compared the clinical presentation and molecular findings of these patients with the previous reports of PHARC syndrome to gain a better realization of the genotype-phenotype correlations of *ABHD12*.

## Methods

### Study participants and clinical evaluations

**Fig. 1:**
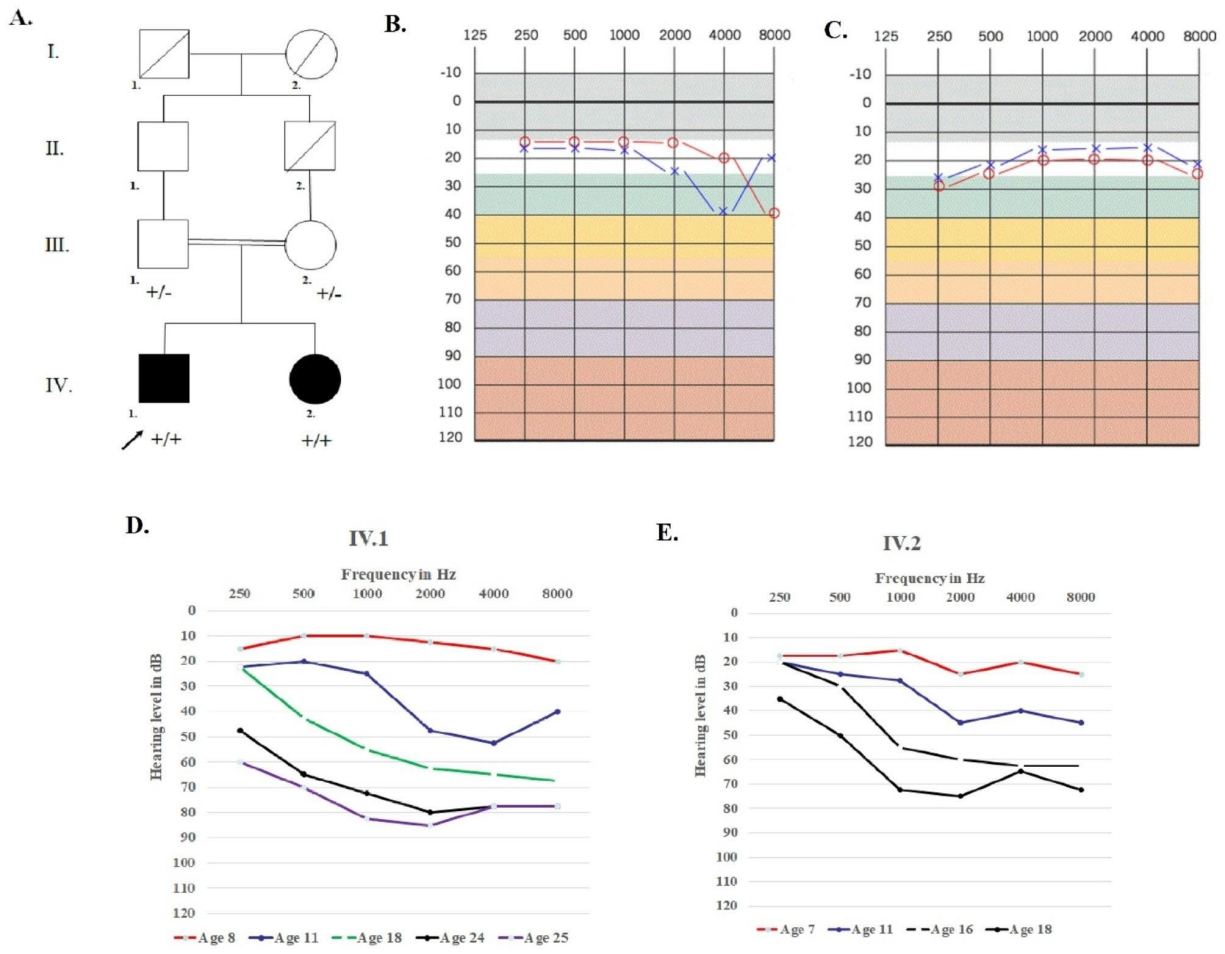
Pedigree information and hearing level in family. **(A)** Pedigree of the family indicates a pattern autosomal recessive inheritance. The pedigree shows co-segregation of *ABHD12* variant ((+) = NM_001042472.3:c.601dup; p.(Val201GlyfsTer4). In this image, the arrow presents proband, black symbols implicate affected; white symbols represent unaffected; circles are females; squares are men; and parallel lines indicate consanguineous marriage. **(B)** Pure tone audiograms of an unaffected father. **(C)** Pure tone audiograms of an unaffected mother **(D)** Audioprofile indicates progressive hearing loss in patient IV.1 in 8-year, 11-year, 18-year, 24-year and 25-year, respectively. **(E)** Audioprofile indicates progressive hearing loss in patient IV.2 in 7-year, 11-year, 16-year and 18-year, respectively. The frequency is shown in hertz (Hz) and the hearing threshold is shown in decibels (dB). The blue ‘×’ and red ‘o’ show results from an air conduction test of the left and right ear, respectively

In this study, two Iranian consanguineous siblings with mild sensory symptoms, progressive hearing impairment, RP, and cataract were enlisted (Fig. 1a). The proband (IV.1) was a 25-year-old male; his 18-year-old sister (IV.2) had the same manifestation but with milder symptoms. Clinical examinations, involving family history and physical exams, were conducted in Hazrat Rasoul Akram Hospital, Tehran, Iran. The patients (IV.1 and IV.2) were examined by otologists, ophthalmologists, and neurologists.

Standard conventional audiometry, including air- and bone-conduction testing, was carried out for IV.1, IV.2, III.1, and III.2 [13]. Additionally, a complete ophthalmologic examination of the afflicted individuals (IV.1 and IV.2) included assessments of best-corrected visual acuity (BCVA), slit lamp bio microscopy, electroretinography (ERG), and optic coherence tomography (OCT). Neurological evaluations include electromyographic recordings, nerve conduction studies comprising measurements of motor and sensory nerves of the upper and lower extremities, and magnetic resonance brain imaging (MRI). Routine laboratory testing was conducted, including tests for liver transaminases, glomerular filtration rate, complete blood count, and electrolytes. Genomic DNA was extracted from blood samples (5 mL) of patients and healthy parents as described before [14].

### Whole-exome sequencing and bioinformatics analysis

WES was done based on the previous works [15, 16]. Briefly, the exomes were captured by the SureSelect Human All Exon V7 Kit (Agilent, Santa Clara, CA, USA). Sequencing was done on an Illumina Hiseq2000 system (Illumina, San Diego, USA) with a mean coverage of 100X. The GRCh38/hg38 genome assembly was used to align reads.

To reach the disease-causing variants, firstly, the variants with minor allele frequency above 1% in databases like bSNP [17], gnomAD [18], and Iranome [19] were removed from the WES data of the patient. Secondly, synonymous changes and all non-coding areas other than the 20 bp flanking regions were eliminated. Bioinformatics techniques such as SIFT [20], Polyphen2 [21], MutationTaster [22], PROVEAN [23], and Combined Annotation Dependent Depletion [24] were used to predict the outcomes of the variants. According to patients’ clinical manifestations (e.g., sensorimotor neuropathy, hearing impairment, and abnormal eye physiology), the remaining variations were prioritized using ClinVar [25], Human Gene Mutation Database (HGMD) [26], human phenotype ontology [27], and Deafness Variation Database (DVD) [28]. Variant interpretation followed the ACMG/AMP (American College of Genetics and Genomics/Association for Molecular Pathology) recommendations [2].

### Family segregation study and protein analysis

Direct Sanger sequencing was used to verify the identified variants in affected members, and co-segregation analysis of the causative homozygous variant was done on all family members. The primers for the area of interest were designed using Primer3 software [29]. The forward primer: 5′-GTCTTTGTCAGGACCCAGGA-3′ and the reverse primer: 5′-AGTCAGGCAGCATGTCACAG-3′ were used to amplify the identified variant in *ABHD12*. PCR was done in standard conditions [15]. The PCR products were used for direct Sanger sequencing and the data were analyzed using Codon code aligner V.5.1.5.

To study the effect of identified mutation on the ABHD12 functional domains ConSurf server (https://consurf.tau.ac.il/) and UniProt [30] were used. Swiss-Model software (https://swissmodel.expasy.org/interactive) was used to design the 3D structure of the protein. I-Mutant3.0 was used to predict protein stability (http://gpcr2.biocomp.unibo.it/cgi/predictors/I-Mutant3.0/I-Mutant3.0.cgi), and MetaDome [31] was used to recognize the intolerant areas in the ABHD12 protein.

### Literature review

In November 2022, a thorough search was conducted in Google Scholar and PubMed using the terms ABHD12 and PHARC syndrome. All original English full-text articles and case reports with clinical and genetic information were added. Available phenotype and genotype were included.

## Results

### Clinical findings

The patients were born to a first-cousin marriage (Fig. 1a). Both patients presented bilateral pes cavus. Audiology evaluations showed a progressive sensorineural hearing impairment in patients (IV.1 and IV.2) that was first distinguished at the age of 11. The audio profiles of patients at different ages are shown in (Fig. 1d, e), and air conduction audiograms of their healthy parents are presented in (Fig. 1b,c).

A physical examination of IV.1 indicated mild symptoms of stance ataxia with positive Romberg and tandem gait signs, while IV.2 was normal. Heel-to-shin and finger-to-nose tests were normal in both patients, and sensory deficits in the sensation of temperature, vibration, and touch could not be found. Furthermore, both patients’ tendon reflexes in the upper and lower extremities were normal, and muscular atrophy and weakness were absent. Routine laboratory tests were normal in both patients.

### Electrophysiology

Both patients’ nerve conduction studies revealed a chronic demyelinating sensorimotor neuropathy with uniform conduction, showing that nerve conduction velocities were well below 40 m/s in both the upper and lower extremities.

Electromyographic recordings in both patients displayed a regular pattern of the motor unit. Pathologic spontaneous activity could not be found.

#### Ophthalmologic examination and brain imaging

**Fig. 2:**
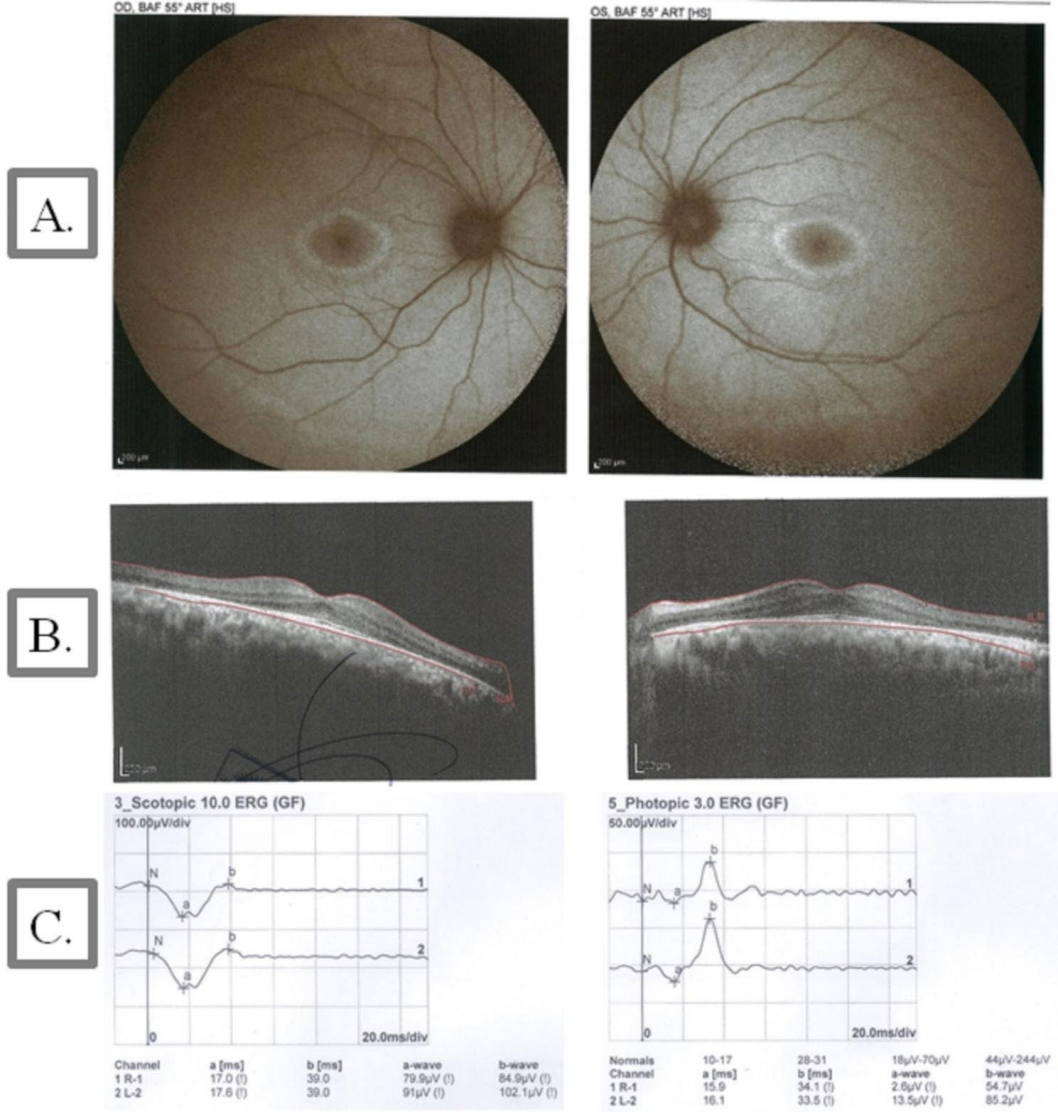
Fundus autofluorescence (FAF), optical coherence tomography (OCT) images, and electroretinography (ERG) in patient IV.1 with *ABHD12* variants. **(A)** The eyes of a 25-year-old man with Snellen best-corrected visual acuity (BCVA) of 2/10. Fundus autofluorescence imaging showed typical ring-shaped macular alterations. **(B)** OCT showed preservation of the outer retinal layers in the fovea with outer retinal atrophy outside fovea. **(C)** ERG revealed a significant reduction in the amplitude of the scotopic and photopic recordings

**Fig. 3:**
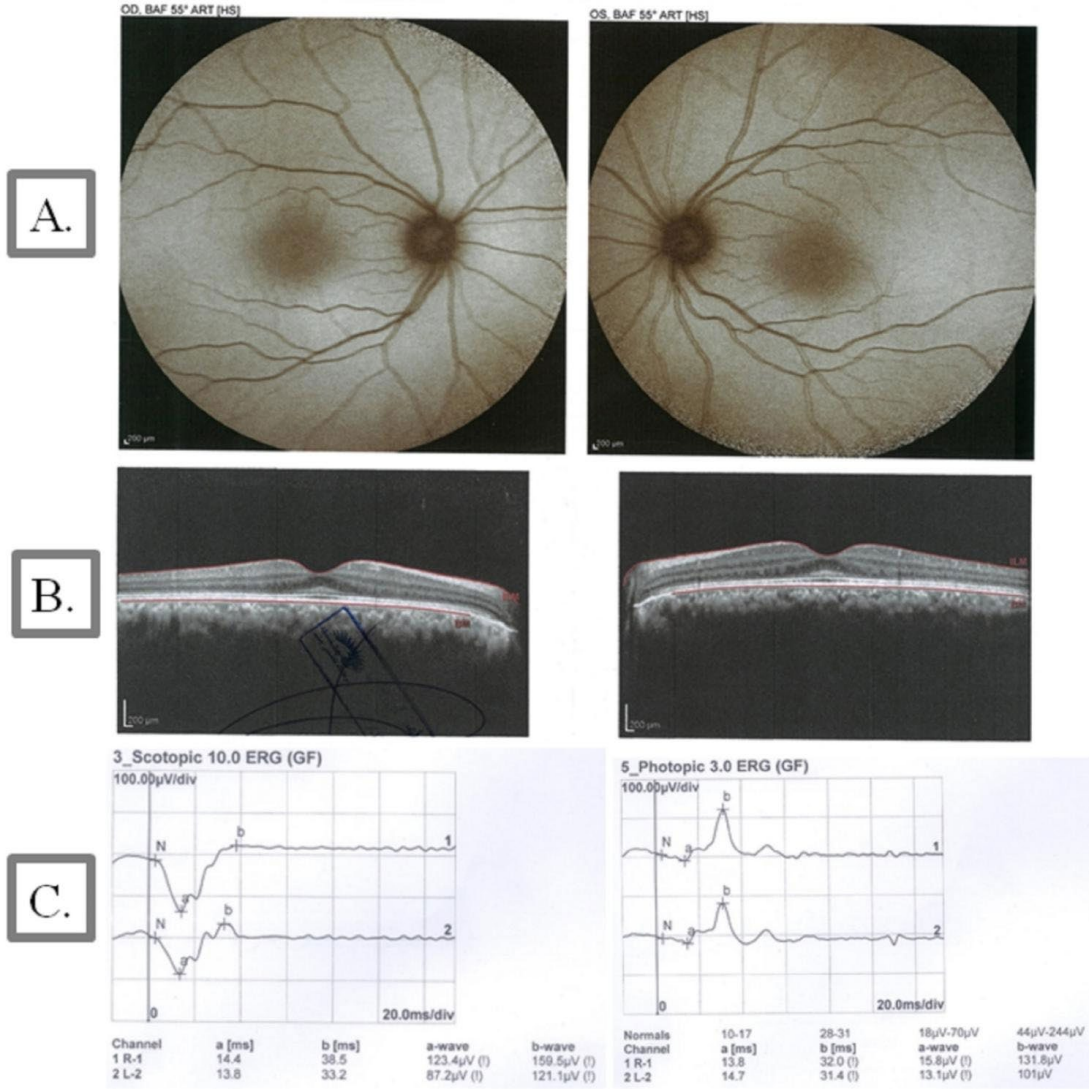
Fundus autofluorescence (FAF), optical coherence tomography (OCT) images, and electroretinography (ERG) in patient IV.2 with *ABHD12* variants. **(A)** The eyes of an 18-year-old woman with Snellen BCVA of 9/10 and 8/10 for the right and left eye, respectively. FAF imaging was preserved in this case. **(B)** On OCT, loss of the ellipsoid zone was observed in the extrafoveal area. **(C)** ERG showed a significant reduction in the amplitude of the scotopic and photopic recordings

**Fig. 4:**
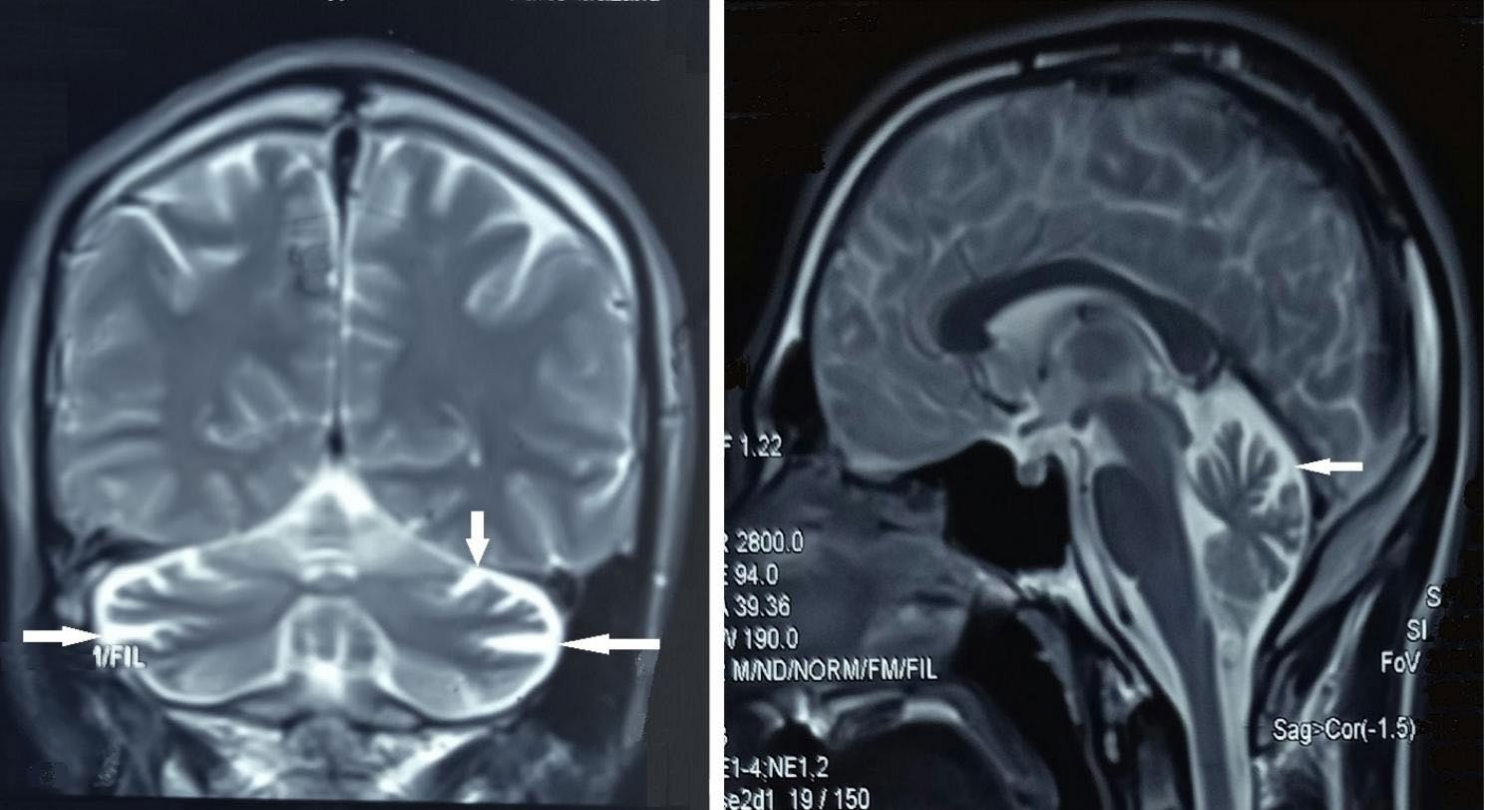
Brain MRI of patient IV.I showing cerebellar atrophy (arrows)

An ophthalmologic examination revealed that BCVA was 2/10 and 2/10 for IV.1 and 9/10 and 8/10 for IV.2 for the right and left eyes, respectively. Patient IV.1 showed a bilateral moderate posterior subcapsular cataract, while his younger sister (IV.2) showed a bilateral mild posterior subcapsular cataract. Both patients showed signs of RP in fundus autofluorescence (FAF), OCT, and ERG (Fig. 2**and** Fig. 3). The MRI of the brain of patient IV.1 revealed cerebellar atrophy (Fig. 4), while it was normal in patient IV.2.

### Molecular findings

**Fig. 5:**
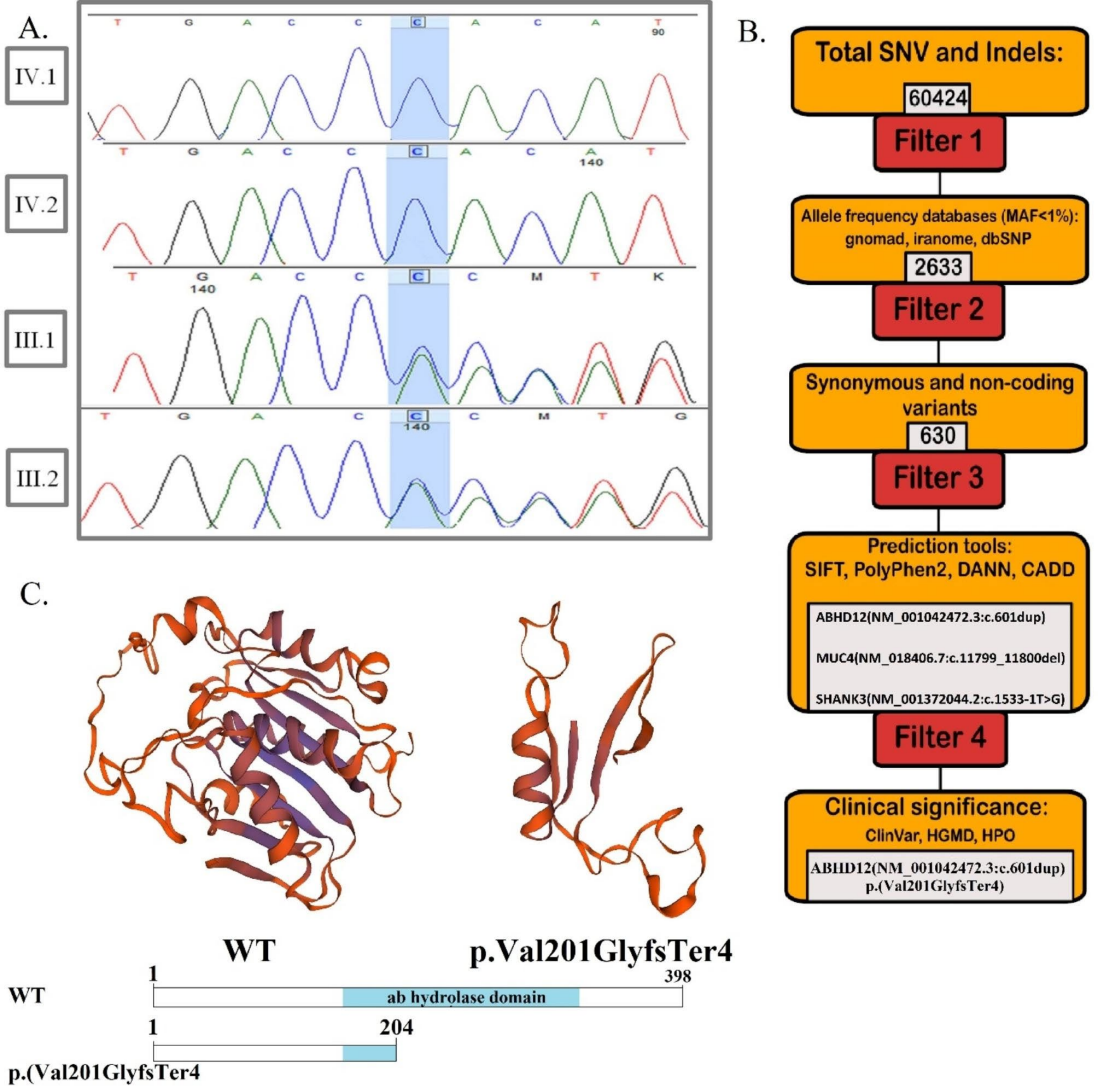
Chromatogram, multiple amino acid alignment, and 3D protein structure. **(A)** The chromatogram for the c.601dup found in the family in exon 6 of *ABHD12* is highlighted in blue. Patient individuals are homozygous (IV.1, IV.2), and their parents are heterozygous (III.1, III.2). **(B)** Schematic representation of filtering strategies used in this study **(C)** The wild-type model structure of ABHD12 protein (left side) and p.Val 201GlyfsTer4 protein (right side). Sequencing analysis showed a novel frameshift variant resulting in premature stop codon of *ABHD12* (bottom side)

Four family members were evaluated in total (Fig. 1a). Firstly, based on the ACMG guidelines for screening for genes associated with hearing loss [2], the absence of mutation in *GJB2* was investigated in both patients (IV.1 and IV.2) [32, 33]. After the analysis of the exome sequencing data on IV.1 (Fig. 5b), a novel frameshift duplication in exon six of the *ABHD12* gene—NM_001042472.3: c.601 dup; p.(Val201GlyfsTer4)— that co-segregated with the phenotype was identified (Figs. 1a and 5a). The variant was not reported in ClinVar, DVD, HGMD, dbSNP v.154, and gnomAD. The allele frequency for this variant was zero in Iranome (local database).

**Fig. 6:**
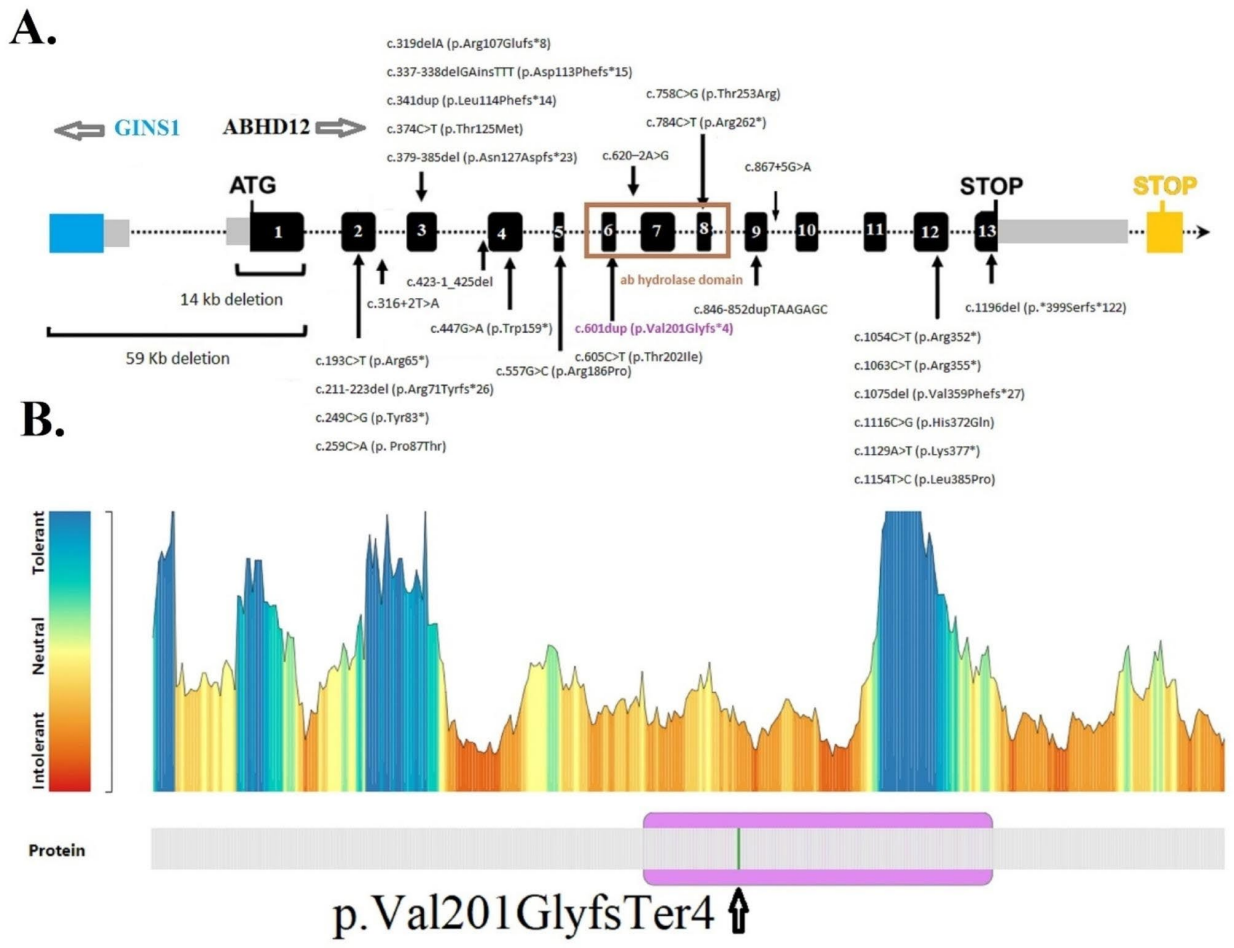
Gene and protein structure. **(A)** Intron-exon structure of *ABHD12* and location of all mutations found up to now. Twenty-nine mutations in *ABHD12* associated with PHARC syndrome have been found. The new frameshift variant, c.601dup, is indicated by the purple color in ab hydrolase domain which is indicated by brown color. Black rectangles and black lines represent exons and introns, respectively. *ABHD12* has 13 exons. The only difference between the two isoforms is in their last exon, which is indicated by two stop codons in the picture (black frame, isoform 1, and orange frame, isoform 2). Gray rectangles indicate 3′UTR and 5′UTR rejoin. The blue rectangle shows GINS1 gene next to *ABHD12* gene. In the 59Kb deletion removes the exon 1 of *ABHD12* and exons 1–4 of GINS1 and both promoters. The gray arrows on the top of the image indicate the orientation of the genes. The figure is redrawn from ref [38] **(B)** MetaDome [31] was used to recognize the intolerant regions in the ABHD12 protein

This variant is located in the αβ-hydrolase domain of the ABHD12 protein (Fig. 6a). We further confirmed this finding by using I-Mutant3.0, which exhibited that this variant can bring the protein close to an unstable (Free Energy change value < − 3.03) and predict its effect on human health (Disease RI: 5). Actually, the I-mutant server calculates the free energy of mutant protein and negative value of free energy change shows a decrease in protein stability. The MetaDome (a server for analysis the mutation tolerance at each position in a human protein) results indicated that this variant was situated in the intolerant regions of the ABHD12 protein (Fig. 6b).

We classified the novel frameshift based on ACMG/AMP guidelines (Criteria: PVS1 and PM2, PM1, PM4, PP3, and PP4) as “pathogenic” variant [2].

### Literature review

A comprehensive analysis of *ABHD12* variants was carried out. Data from this research were compared with 14 previously published articles [7–9, 11, 12, 34–42]. In summary, 58 patients from 38 families were included. 29 distinct *ABHD12* mutations have been identified in these published articles. Their phenotype, genotype, age, and sex are summarized in (Table 1), while all variants are illustrated in Fig. 6. It has been documented that ABHD12 exhibits a broad range of clinical heterogeneity in terms of age of onset, spectrum of phenotypes, severity, and progression. Cataract and hearing impairment were the most common conditions reported in ABHD12 patients.

**Table 1:** Summary of the reported *ABHD12* mutations in associated with PHARC syndrome

Nucleotide change*	AA change	E I	Mutation type	Age Sex	Poly neuropathy	Motor Neuropathy	HL	RP	ERG	Cataract	Ataxia	MR CT	Pyramidal Tract Signs	Other	Country	Family case	Ref
14 kb deletion removing exon 1		E1	Deletion	24 M	Abnormal	Pes cavus; absent tendon reflexes	14y Deaf	20y	NR	15y	Mild	Normal	Indifferent plantar response	No	UAE	6.1	[7]
14 kb deletion removing exon 1		E1	Deletion	20 M	Demyelinating polyneuropathy	Pes cavus; absent tendon reflexes	6y	Yes	NR	Yes	Speech and limb ataxia; wheelchair-bound (10y)	Cerebellar atrophy 3y	Extensor plantar response	No	UAE	6.2	[7]
14 kb deletion removing exon 1		E1	Deletion	6 F	NR	Absent tendon reflexes	Yes	No	NR	Yes	Speech and limb	Cerebellar atrophy	Indifferent plantar response	No	UAE	6.3	[7]
c.193 C > T	p.Arg65*	E2	Nonsense	55 F	No	No	17y 35y CI	Yes	Rod-cone abnormal	Yes	Ataxic gait with poor tandem walking; action tremor with writing cramp and involuntary athetotic movements of her fingers	Normal	NR	Night blindness, glaucoma	Lebanese	II.1	[41]
c.193 C > T	p.Arg65*	E2	Nonsense	53 M	NR	NR	24y 35y CI	Yes	NR	Posterior subcapsular cataract	NR	NR	NR	Glaucoma; severe optic atrophy	Lebanese	II.4	[41]
c.193 C > T	p.Arg65*	E2	Nonsense	22 M	Yes	7y; Lack of coordination	No	Yes	Rod-cone dystrophy	No	NR	NR	NR	No	NR	ABHD12-3 H-10	[34][8]
c.211-223del	p.Arg71Tyrfs*26	E2	Frameshift	42 M	Demyelinating polyneuropathy	Pes cavus; hammertoes; sensory loss;	43y bilaterally CI	Yes	Rod-cone dystrophy	38y	Wide-base gait, action tremor in the upper limbs	Mild cerebellar atrophy	NR	muscle MRI revealed mild fatty infiltration in the intrinsic muscles of both feet and the anterior compartment of the left leg	Spanish	II.3	[37]
c.211-223del	p.Arg71Tyrfs*26	E2	Frameshift	NR M	Demyelinating polyneuropathy	Pes cavus; hammertoes; sensory loss	18y	Yes	NR	Yes	Wide-base gait; dysarthria; slight dysmetria	Mild cerebellar atrophy	NR	No	Spanish	II.1	[37]
c.249 C > G	p.Tyr83*	E2	Nonsense	41 M	NR	NR	32y progressive	Yes	Abnormal	Yes	NR	NR	NR	Olfactory decline	China	I.1	[36]
c.249 C > G	p.Tyr83*	E2	Nonsense	31 M	NR	NR	32y progressive	Yes	NR	Yes	NR	NR	NR	Olfactory decline	China	II.5	[36]
c.259 C > A c.1063 C > T	p. Pro87Thr p.Arg355*	E2 E12	Missense Nonsense	28 F	NR	NR	No	Yes	Rod-cone dystrophy	No	No	NR	NR	No	NR	ABHD12-2	[34]
c.316 + 2T > A		I2	Splice site	56 M	Yes	Sensory loss	15y 41y CI	22y	NR	Yes	Steppage gait	Normal	NR	No	Japan	KTM 5012	[39]
c.316 + 2T > A		I2	Splice site	64 M	NR	NR	Progressive	45y	NR	Yes	NR	Cerebral and cerebellar atrophy	NR	Epilepsy; 30y night blindness	Japan	SNS5547	[39]
c.316 + 2T > A		I2	Splice site	NR M	NR	NR	Deaf	Yes	NR	Yes	NR	NR	NR	Epilepsy	Japan	SNS5548	[39]
c.319delA c.605 C > T	p.Arg107Glufs*8 p.Thr202Ile	E3 E6	Frameshift Missense	78 F	No	Decreased vibration sense in lower limbs	Presbycusis	Yes	NR	Yes	No	Minimal cerebral atrophy	No	No	Spain	1292-II.4	[40]
c.319delA c.605 C > T	p.Arg107Glufs*8 p.Thr202Ile	E3 E6	Frameshift Missense	75 M	No	Slightly loss of strength right upper limb	Presbycusis	Yes	NR	Yes	Dysmetria in lower limbs; slightly wide basegait	Normal	No	No	Spain	1292-II.5	[40]
c.319delA c.605 C > T	p.Arg107Glufs*8 p.Thr202Ile	E3 E6	Frameshift Missense	72 F	No	No	NR	Yes	NR	Yes	Gait somewhat unstable	NR	No	No	Spain	1292-II.6	[40]
c.319delA c.605 C > T	p.Arg107Glufs*8 p.Thr202Ile	E3 E6	Frameshift Missense	66 M	No	No	Presbycusis	Yes	Diminished	Yes	Slight dysarthria; mild distal postural tremor	Normal	No	No	Spain	1292-II.7	[40]
c.337-338delGAinsTTT	p.Asp113Phefs*15	E3	Frameshift	62 F	Demyelinating polyneuropathy	38y; pes cavus; sensory loss; absent ankle reflexes	20y	38y	Rod-cone dystrophy	28y	No	Normal	No	No	Norway	1.1	[7]
c.337-338delGAinsTTT	p.Asp113Phefs*15	E3	Frameshift	56 M	Demyelinating polyneuropathy	37y; pes cavus	30y	37y	Rod-cone dystrophy	37y	37y; gait ataxia	Normal	Extensor plantar response at lower limbs; spasticity; hyperreflexia	No	Norway	1.2	[7]
c.337-338delGAinsTTT	p.Asp113Phefs*15	E3	Frameshift	46 M	Demyelinating polyneuropathy	38y; distal sensory loss	Yes	46y	Rod-cone dystrophy	25y	43y; gait ataxia; upper limb intention tremor	Cerebellar atrophy	Extensor plantar response at lower limbs; spasticity; hyperreflexia	No	Norway	1.3	[7]
c.337-338delGAinsTTT	p.Asp113Phefs*15	E3	Frameshift	58 M	Demyelinating/ axonal polyneuropathy	51y; pes cavus; sensory loss; reduced tendon reflexes	20y	35y	Rod-cone dystrophy	26y	No	Cerebellar atrophy	Extensor plantar response at lower limbs	No	Norway	2.1	[7]
c.337-338delGAinsTTT	p.Asp113Phefs*15	E3	Frameshift	54 F	Yes	53y; pes cavus; reduced tendon reflexes	20y	25y	Flat	25y	No	NR	No	NO	Norway	2.2	[7]
c.337-338delGAinsTTT	p.Asp113Phefs*15	E3	Frameshift	36 F	Demyelinating polyneuropathy	Pes cavus; reduced tendon reflexes in lower limbs	10y Deaf	36y	Rod-cone dystrophy	32y	Yes	Atrophy of vermis and medulla oblongata	Extensor plantar response at right side; spasticity	No	Norway	3.1	[7]
c.337-338delGAinsTTT	p.Asp113Phefs*15	E3	Frameshift	24 M	Demyelinating polyneuropathy	Pes cavus; hammertoes; reduced tendon reflexes in upper and lower limbs	Yes	No	Normal	15y	No	Slight ventricular asymmetry	Indifferent plantar response	No	Norway	4.1	[7]
c.337-338delGAinsTTT	p.Asp113Phefs*15	E3	Frameshift	16 M	Demyelinating polyneuropathy	Pes cavus; reduced sensibility; reduced tendon reflexes in upper limbs, absent in lower limbs	13y	No	Normal	16y slight	No	Normal	No	No	Norway	5.1	[7]
c.337-338delGAinsTTT	p.Asp113Phefs*15	E3	Frameshift	20 M	Yes	NR	16y	Yes	NR	Star-shaped cataract 17y	No	Cerebellar atrophy	NR	No	NR	J-12	[8]
c.337-338delGAinsTTT	p.Asp113Phefs*15	E3	Frameshift	17 M	Yes	NR	10y	Yes	NR	Star-shaped cataract 10y	No	Cerebellar atrophy	NR	No	NR	J-13	[8]
c.337-338delGAinsTTT	p.Asp113Phefs*15	E3	Frameshift	36 F	NR	NR	12y	Yes	Rod-cone dystrophy	Posterior subcapsular cataract 32y	Yes	NR	NR	No	NR	E-7	[8]
c.337-338delGAinsTTT	p.Asp113Phefs*15	E3	Frameshift	32 F	Yes	NR	17y	Yes	Rod-cone dystrophy	Posterior subcapsular cataract 32y	45y	NR	NR	No	NR	B-2	[8]
c.337-338delGAinsTTT c.341dup	p.Asp113Phefs*15 p.Leu114Phefs*14	E3 E3	Frameshift Frameshift	39 M	NR	NR	33y	Yes	Rod-cone dystrophy	Star-shaped cataract 29y	NR	NR	NR	No	NR	L-15	[8]
c.337-338delGAinsTTT c.423-1_425del	p.Asp113Phefs*15 p.(?)	E3 I3	Frameshift Splice site	33 M	Yes	Subtle foot drop; absent Achilles tendon reflexes	No	Yes	NR	Sutural cataract 3y	27y	Normal	NR	No	NR	C-3	[8]
c.337-338delGAinsTTT c.423-1_425del	p.Asp113Phefs*15 p.(?)	E3 I3	Frameshift Splice site	33 M	Yes	Distal muscle weakness; sensory loss	Yes	Yes		Sutural cataract 3y	27y	Normal	NR	No	NR	C-4	[8]
c.337-338delGAinsTTT c.423-1_425del	p.Asp113Phefs*15 p.(?)	E3 I3	Frameshift Splice site	38 M	Yes	Abnormal gait pattern; distal sensory loss	20y	Yes		Star-shaped cataract 4y	31y	Cerebellar atrophy	NR	No	NR	C-5	[8]
c.337-338delGAinsTTT c.1075del	p.Asp113Phefs*15 p.Val359Phefs*27	E3 E12	Frameshift Frameshift	47 M	Yes	Pes cavus, hammertoes, distal sensory loss, absent tendon reflexes 8y	28y	Yes	Rod-cone dystrophy	36y	8y	Normal	NR	No	NR	A-1	[8]
c.374 C > T c.1154T > C	p.Thr125Met p.Leu385Pro	E3 E12	Missense Missense	53 M	NR	NR	44y progressive	Yes	NR	Posterior polar cataract 41y	NR	NR	NR	Epilepsy; learning difficulties	NR	ABHD12-5 I-11	(8, 34)
c.379-385del AACTACT ins GATTCCTTATAT ACCATTGTAGTC TTACTGCTTTTG GTGAACACA	p.Asn127Aspfs*23	E3	Deletion-Insertion	36 M	Demyelinating polyneuropathy	Yes	5y Deaf	No	NR	28y	15y ataxic walk	Normal	NR	No	France	XIX.1	[12]
c.447G > A c.557G > C	p.Trp159* p.Arg186Pro	E4 E5	Nonsense Missense	30 M	Yes	Distal sensory loss; reduced tendon reflexes	Yes	Yes	Rod-cone dystrophy	Cortical cataract	Wide-based gait; Stuttering speech; ataxic gait	Normal	No	No	Netherlands	W08-1833 D-6	(8, 40)
c.601dup	p.Val201Glyfs*4	E6	Frameshift	25 M	Yes	Pes cavus; slight gait disturbance	Yes	Yes	Abnormal	Yes	Yes	Cerebellar atrophy	No	No	Iran	IV.1	This study
c.601dup	p.Val201Glyfs*4	E6	Frameshift	18 F	Yes	Pes cavus	Yes	Yes	Abnormal	Yes	No	Normal	No	No	Iran	IV.2	This study
c.620–2 A > G		I6	Splice site	34 M	Yes	31y; Lower limb muscle weakness	20y progressive	Yes	Rod-cone dystrophy	26y	No	NR	NR	No	NR	ABHD12-4 G-9	(8, 34)
c.758 C > G	p.Thr253Arg	E8	Missense	31 F	Demyelinating polyneuropathy	Sensory loss	31y bilateral CI	Yes	NR	Posterior subcapsular cataract8y	ataxia 16y; mild intentional tremor; mild dysarthria; wheelchair-bound	Normal	NR	Decreased function of the labyrinths	Swedish	II.1	[38]
c.784 C > T c.867 + 5G > A	p.Arg262*	E8 I9	Nonsense Splice site	53 M	Yes	53y; Distal sensory loss	20y progressive	Yes	NR	No	No	NR	NR	No	NR	ABHD12-6 F-8	(8, 34)
c.784 C > T	p.Arg262*	E8	Nonsense	21 M	Demyelinating polyneuropathy	Pes cavus: reduced tendon reflexes at upper and lower limbs	9y 17y CI	No	NR	Yes	Stance ataxia	Cerebellar atrophy	No	No	Iraq	A	[35]
c.784 C > T	p.Arg262*	E8	Nonsense	25 M	Demyelinating polyneuropathy	Sensory loss; Loss of Achilles and patellar tendon reflexes	12y 18y CI	Yes	NR	Yes	Slight bilateral limb ataxia	NR	NR	No	Iraq	B	[35]
c.846-852dupTAAGAGC	p.His285fs*1	E9	Frameshift	11 M	Yes	Absent tendon reflexes; moderate muscle weakness at lower limbs	No	No	NR	No	3-4y; limb ataxia ; horizontal nystagmus; dysarthria; dysmetria delayed walking at 15 months; action and intention tremor	Cerebellar atrophy	Extensor planter response at lower limbs	No	Algeria	8.1	[7]
c.846-852dupTAAGAGC	p.His285fs*1	E9	Frameshift	10 F	Yes	Absent tendon reflexes at lower limbs	No	No	NR	No	4-5y; gait ataxia	Vermian atrophy	Extensor planter response at lower limbs	No	Algeria	8.2	[7]
c.846-852dupTAAGAGC	p.His285fs*1	E9	Frameshift	44 M	Demyelinating polyneuropathy	Pes cavus; sensory loss; absent tendon reflexes at lower limbs; scoliosis	Yes	Amblyopia	NR	NR	7-10y limb ataxia; dysarthria; dysmetria at upper limbs with adiadocokinesia; head titubation	Vermian atrophy	Extensor planter response at lower limbs; macroglossia	No	Algeria	9.1	[7]
c.846-852dupTAAGAGC	p.His285fs*1	E9	Frameshift	26 F	Demyelinating polyneuropathy	Pes cavus; sensory loss; reduced tendon reflexes at upper limbs, and absent at lower limbs; tongue fasciculation	Deaf	Yes	NR	Yes	4-9y gait and limb ataxia; horizontal nystagmus; moderate dysarthria; dysmetria at upper and lower limbs	Vermian atrophy	Extensor planter response at lower limbs;	No	Algeria	9.2	[7]
c.846-852dupTAAGAGC	p.His285fs*1	E9	Frameshift	26 F	Severe demyelinating polyneuropathy on nerve biopsy	Pes cavus; sensory loss; absent tendon reflexes	6y	No	NR	No	6-12y limb ataxia	Normal	Indifferent plantar response	No	Algeria	10.1	[7]
c.846-852dupTAAGAGC	p.His285fs*1	E9	Frameshift	19 F	Yes	12y; pes cavus; sensory loss; absent tendon reflexes at upper and lower limbs	NR	NR	NR	NR	No	NR	NR	No	Algeria	10.2	[7]
c.846-852dupTAAGAGC	p.His285fs*1	E9	Frameshift	32 F	Axonal polyneuropathy	Pes cavus; sensory loss; absent tendon reflexes at lower limbs	Yes	Decreased visual acuity and amblyopia	NR	No	16-20y; gait ataxia; dysarthria; dysmetria at upper limbs	Cerebellar atrophy	Extensor plantar response at lower limbs	No	Algeria	11.1	[7]
c.846-852dupTAAGAGC	p.His285fs*1	E9	Frameshift	23 F	Yes	NR	Yes	Yes	NR	Yes	Yes	NR	NR	No	Spain	10	[42]
c.1054 C > T	p.Arg352*	E12	Nonsense	50 F	Abnormal	34y; pes cavus; hammertoes;	17y	20y	NR	22y	18y; dysarthria; gait ataxia; jerky eye movement tremor in hand	Cerebellar atrophy Increased signal in periventricular white matter	Flexor planter response; spasticity;	No	USA	7.1	[7]
c.1054 C > T	p.Arg352*	E12	Nonsense	29 F	Demyelinating polyneuropathy	Pes cavus; sensory loss; absent tendon reflexes; a mild waddling	16y	Yes	NR	Yes	Gait ataxia; progressive jerky tremor; mild titubation of the trunk; mild proximal tetraparesis; dysmetria; mild bilateral ptosis; congenital convergent strabismus	Global moderate cerebral/cerebellar atrophy	NR	Mild learning disabilities	Portugal		[9]
c.1054 C > T c.1196del	p.Arg352* p.*399Serfs*122	E12 E13	Nonsense	48 F	NR	NR	No	yes	No Rod-cone dystrophy	Yes	No	NR	NR	No	NR	ABHD12-1	[34]
c.1063 C > T	p.Arg355*	E12	Nonsense	46 F	Yes	NR	Yes	Yes	Rod-cone dystrophy	Cerulean cataract	Yes	NR	NR		NR	K-14	[8]
c.1116 C > G	p.His372Gln	E12	Missense	38 F	Demyelinating polyneuropathy	Distal symmetric hypoesthesia to touch and pain; achilles reflex abolished	38y	Yes	Diminished	Yes	No	Cerebral and cerebellar atrophy	NR	No	Spain	RP-1487	[40]
c.1129 A > T 59 Kb deletion including exon 1	p.Lys377*	E12 E1	Nonsense Deletion	29 F	Demyelinating polyneuropathy	Pes cavus; sensory loss; absent tendon reflexes	HL from Childhood 21y bilateral CI	Yes	Abnormal	Yes	Steppage gait; mild dysmetria	Normal	Flexor plantar response	No	Japan	III.1	[11]

Abbreviations: AA, amino acid; E, exon; I, Intron; HL, hearing loss; RP, retinitis pigmentosa; ERG, electroretinography; MR, magnetic resonance; CT, computed tomography; Family case; the patients ID in relevant article; Ref, reference; M, male; F, female; Y, year; CI, cochlear implant; NR, not reported in article. * Nucleotide numbering is based on reference sequence NM_001042472.3.

Most mutations reported in *ABHD12* were frameshift mutations (Table 1). The c.337-338delGAinsTTT is the most common variant. only seven of these variants (c.193 C > T, c.316 + 2T > A, c.337-338delGAinsTTT, c.784 C > T, c.846-852dupTAAGAGC, c.1054 C > T, and c.1063 C > T) have been reported in more than one family.

## Discussion

In this study, we detected a novel frameshift variant in the *ABHD12* gene in two affected Iranian siblings with PHARC syndrome from a first-cousin marriage (Fig. 1a). The identified variant, c.601dup; p.(Val201GlyfsTer4), leads to a premature stop codon (Fig. 5c), which can result in a loss of function, and was determined as a pathogenic variant in agreement with ACMG guidelines [2].

PHARC syndrome is distinguished by hearing impairment, polyneuropathy, RP, ataxia, and early-onset cataract. The variety of clinical symptoms showed that ABHD12 play crucial roles in the in the central and peripheral nervous systems, as well as the eye, which is confirmed by its expression patterns [7]. *ABHD12* is expressed ubiquitously and is extremely expressed in the brain, especially in microglia, macrophages, and in the retina [7, 43].

*ABHD12* was detected on chromosome 20 (20p11.21) for the first time in 2010. 29 mutations in 58 patients (38 families) from 14 previously published articles related to the PHARC syndrome around the world have been introduced (Table 1; Fig. 6a). These patients exhibited clinical variability concerning the spectrum of phenotypes, disease onset, severity, and progression [7, 8], and this variability was observed both within the same family and between patients with the same variant from different families (Table 1) [8, 37]. In addition, there is no correlation between the location and type of mutation and the severity of phenotypes in patients. For example, in a comparison between two nonsense mutations (p.Arg352* and p.Arg65*), the patients with the first mutation in early adulthood showed complete phenotypes of PHARC syndrome, while the patients with the second mutation did not experience neuropathy until the fifth decade of their lives (Table 1) [41]. The current evidence does not indicate any genotype-phenotype correlation in patients with mutations in the *ABHD12* gene. However, the limited number of reported cases, the multisystemic nature of the PHARC syndrome (which leads to misdiagnosis or delayed diagnosis), delayed referral for evaluation of related phenotypes, or failure to record all phenotypes at the same time in different studies can be effective.

This study’s proband (IV.1) manifested a typical PHARC phenotype, the onset of which dates to the patient’s early teenage years. It had a progressive nature, eventually revealing hearing impairment, bilateral posterior subcapsular cataract, ataxia, demyelinating polyneuropathy, and RP. The clinical picture was completely compatible with PHARC syndrome when the patient was 24 years old. The progression of the disease in the second affected family member was the same, though the symptoms were milder.

In line with most previous studies, sensorineural hearing impairment was the first manifestation in both patients (Table 1). Figure 1 indicates the progress of hearing impairment in both patients. Both patients developed posterior subcapsular cataract during childhood, corroborating previous reports showing that posterior subcapsular cataract frequently occurs in RP patients at a young age [44]. Similar to previous studies, our patients’ definitive diagnosis of PHARC syndrome after a long follow-up period was possible only using WES [7, 8, 35]. The multisystemic nature and slow progression of PHARC syndrome is the main reason for its misdiagnosis. Performing genetic testing next to clinical findings could lead to early diagnosis, timely referrals, and better management of future symptoms.

The *ABHD12* gene encodes a 398-amino acid protein product that participates in endocannabinoid metabolism and synaptic plasticity. This product is called the ABHD12 protein, which is a member of the serine hydrolase family and inactivates the endocannabinoid neurotransmitter 2-AG [35, 38]. Furthermore, previous in vivo studies indicated the lysophosphatidylserine (LPS) lipase activity of *Abhd12* in the mouse brain and the accumulation of LPS in the mouse model. This accumulation increases phagocytosis activity and microglial activation, which causes neuroinflammation and atrophy in the cerebellum. This neuroinflammation is the presumed cause of motor and auditory defects over time [45–47].

ABHD12 is a single-pass integral membrane protein with a transmembrane helix in the N-terminal region and an extracellular active site domain in the C-terminal region [48]. The αβ-hydrolase domain of ABHD12 consists of a lipase motif and catalytic triad (predicted amino acid residues S246-D333-H372), which serves as a fully conserved structure in both humans and rodents [49]. This domain expands between residues 165–351 of ABHD12 (Fig. 6) [36]. The p.(Val201GlyfsTer4) variant occurs within the conserved αβ-hydrolase domain and causes a premature stop codon, which may result in nonsense-mediated decay and, consequently, a lack of the protein product. Navia-Paldanius et al. have shown that site-directed mutagenesis of residues of the catalytic triad of the αβ-hydrolase domain abolished the enzymatic activity of ABHD12 [49]. The research group of Tingaud-Sequeira et al. with functional studies on p.R352* mutation that produces a truncated protein have proved the loss of enzyme activity [38]. Moreover, the variants in this domain are likely to disturb interactions with other molecules or other parts of the protein and affect protein function [38].

ABHD12 is a critical protein in the signaling, metabolism, and regulation of lipids, especially in immune and neurological processes [8, 38, 45, 47].

However, further research is required to fully understand the cellular, molecular, and biochemical mechanisms through which ABHD12 contributes to the PHARC syndrome. Such research could lead to earlier diagnosis, appropriate referrals, effective prognosis for future rehabilitations, improved medical management of disease progression, better genetic counseling, and prevention strategies, and a higher increasing quality of life for patients and their relatives.

A significant limitation in this research pertains to the inability to perform a functional analysis that would elucidate the specific contribution of the newly identified variant to PHARC syndrome.

## Conclusion

We elucidated the role of a novel pathogenic mutation in the *ABHD12* as a genetic reason of PHARC syndrome in an Iranian family. Additionally, we demonstrated the value of using WES for the early diagnosis of this syndrome. Our findings extend the mutation spectrum of *ABHD12* by introducing a novel mutation. We also summarized previously reported mutations in the *ABHD12* gene throughout the world and compared them to the new mutation investigated in the present study. We believe these results can help practitioners identify disease pathology and manage the phenotypes in a multidisciplinary setting.

## Data Availability

The datasets produced during this manuscript are available from the corresponding author upon reasonable request. The novel variant and phenotypes were submitted in ClinVar database (accession number: VCV001727244.1) and available at (https://www.ncbi.nlm.nih.gov/clinvar/variation/1727244/).

## Abbreviations

PHARC: polyneuropathy hearing loss ataxia retinitis pigmentosa cataracts
RP: Retinitis pigmentosa
ABHD12: α/βhydrolase domain-containing 12
2-AG: 2-arachidonoyl glycerol
WES: whole-exome sequencing
BCVA: best-corrected visual acuity
ERG: electroretinography
OCT: optic coherence tomography
MRI: magnetic resonance brain imaging
Val: valine
Gly: glycine
ACMG/AMP: American College of Medical Genetics/Association for Molecular Pathology
